# A Bilayer Vaginal Tablet for the Localized Delivery of Disulfiram and 5-Fluorouracil to the Cervix

**DOI:** 10.3390/pharmaceutics12121185

**Published:** 2020-12-06

**Authors:** Ismin Zainol Abidin, Emanuele Rezoagli, Bianca Simonassi-Paiva, Gustavo Waltzer Fehrenbach, Kevin Masterson, Robert Pogue, Zhi Cao, Neil Rowan, Emma J. Murphy, Ian Major

**Affiliations:** 1 Materials Research Institute, Athlone Institute of Technology, Dublin Road, N37 HD68 Athlone, Ireland; i.izabidin@research.ait.ie (I.Z.A.); zcao@research.ait.ie (Z.C.); 2 Bioscience Research Institute, Athlone Institute of Technology, Dublin Road, N37 HD68 Athlone, Ireland; emanuele.rezoagli@unimib.it (E.R.); biancasimonassi@gmail.com (B.S.-P.); gfehrenbach@research.ait.ie (G.W.F.); k.masterson@research.ait.ie (K.M.); repogue@gmail.com (R.P.); nrowan@ait.ie (N.R.); emurphy@ait.ie (E.J.M.); 3 Department of Medicine and Surgery, University of Milan-Bicocca, 1–20126 Monza, Italy; 4 Regenerative Medicine Institute (REMEDI) at CÚRAM Centre for Research in Medical Devices, School of Medicine, National University of Ireland Galway, H91 TK33 Galway, Ireland; 5 Post-Graduate Program in Genomic Sciences and Biotechnology, Catholic University of Brasilia, Brasilia 70790-160, Brazil

**Keywords:** chitosan, polyacrylic acid, mucoadhesion, cervical cancer, vaginal drug delivery, bilayer tablets, combination therapy, polyelectrolyte complex

## Abstract

This study was performed to develop an adjuvant therapy in the form of a self-administered vaginal tablet regimen for the localized delivery of chemotherapeutic drugs. This therapy will help to reduce relapse by eradicating cancerous cells in the margin of cervical tumors. The vaginal tablet is a very common formulation that is easy to manufacture, easy to place in the vagina, and has a low cost of manufacture, making them ideal for use in developing countries. A combination of disulfiram and 5-fluorouracil, which are both off-patent drugs and provide different modes of action, were evaluated. The tablets developed were evaluated for weight variation, thickness, hardness, friability, swelling index, differential scanning calorimetry (DSC), particle morphology, in vitro drug release, and cytotoxicity on Ca-Ski cells. Both layers were designed to release both drugs concurrently for a synergistic effect. The polymer–polymer interaction between the layers was able to reduce the loss of formulation due to chitosan. While the bilayer tablet had satisfactory performance in the physicochemical tests, in vitro cell culture with Ca-Ski also showed a synergistic effect using a combination of drugs at a low dose. However, the formulation only had 24-h dose release before degradation. Further drug combinations should be evaluated in subsequent studies.

## 1. Introduction

The vaginal administration of various agents has several advantages over alternatives, such as the presence of rich blood supply and large surface area of the vagina, avoidance of the hepatic first-pass metabolism, possible self-insertion and removal of the dosage form, and the ability to achieve high local drug concentration [1]. The vagina has been used to deliver drugs for a range of clinical and research applications, including contraception, vaginal infections, and HIV (human immunodeficiency virus) prevention, with many different vaginal formulations, such as gels, creams, pessaries, suppositories, diaphragms, rings, films, tablets, and capsules. Furthermore, a number of these delivery systems have been investigated for the localised delivery of chemotherapeutic drugs to the cervix [2]. The localized delivery of chemotherapeutic drugs offers a number of advantages over systemic administration, including: (1) direct delivery to the site of action, (2) a lower required dose, (3) limiting systemic drug toxicities, and (4) increased drug stability as it remains in the delivery device until released [2].

Cervical cancer is a cancer of the cells located in the cervix [3]. It is the third most common cancer in women globally and is mainly caused by the sexual transmission of the human papillomavirus (HPV) [2,3,4,5,6]. The location of the cervix makes it easily accessible through the vagina and allows for a non-invasive localised delivery of chemotherapeutic drugs; adjacent to the cancerous tissue either before resection (neoadjuvant therapy), to reduce tumour size, or after resection (adjuvant therapy) to reduce the risk of recurrence [7]. The use of local drug delivery systems for the treatment of metastatic cervical tumours may be inefficient when the disease is disseminated in distant organs, such as the lungs, in which case there may be a need for a more systemic approach. However, recent reports show that less than 20% of the cases appear with distant metastasis, which emphasizes that most cervical cancer patients would benefit from localised drug delivery systems [8].

Each year, approximately 0.6 million women are diagnosed and almost 0.3 million deaths are attributed to cervical cancer [9,10]. More than 85% of that burden occurs in women living in low resource countries (developing countries), especially African countries, due to the lack of successful prevention and control programs against this disease [11]. Alternative screening methods and treatments have been adopted to compensate for the lack of resources. Cervical cancer precursors are screened using visual inspection tests either in combination with 3–5% acetic acid (VIA) or Lugol’s iodine (VILI). The results are instantaneous and women who screen positive for precancerous lesions will be offered a treatment during the same visit [12]. The most common treatment offered is cryotherapy, which is an ablation (destruction) method involving freezing the affected tissue. This screen and treat strategy can be performed at the primary care level by secondary healthcare workers with minimum supplies and equipment [12,13]. Despite the limitations of this concept, it helps to overcome barriers of time, distance, and loss to follow-up [11].

Our contention is that a vaginal tablet chemotherapeutic formulation can be developed that would complement this current treatment strategy. After surgical treatment the patient will be supplied with a course of these vaginal tablets that can be easily self-administered. Similar to tablets intended for other administration routes, vaginal tablets show advantages including precise dosing, better drug stability, avoidance of antimicrobial agents for preservation, easier handling and storage, and low cost due to large scale production. Furthermore, self-administration is quite easy with no applicators or supervision needed [14]. We developed a profile for this intervention that consists of a number of key criteria: (1) drug selection, (2) polymer selection, and (3) tablet manufacturing.

(1) Drug selection: A simple criteria for active drug selection was established that would be best suited for deployment, namely being low-cost and off-patent. As an antimetabolite drug, 5-fluorouracil (5-FU) has two mechanisms of action: (i) inhibition of the thymidylate synthase enzyme and (ii) misincorporation into DNA and RNA [2]. It was developed more than 50 years ago and continues to be widely used in the treatment of cancer [15,16]. The topical formulation of 5-FU is used to treat skin cancer and HPV-related warts, lesions, and neoplasia [2]. Moreover, 5-FU has a relatively short half-life [17], which requires frequent doses. Dose-related side effects include severe nausea and vomiting, pain, and chronic ulceration. Studies have shown that less frequent or diluted doses have reported favourable side effect profiles [18]. Therefore, to reduce the amount of 5-FU required, a co-drug was incorporated to produce a synergistic effect and increase safety and the efficacy of the formulation [19]. One approach to expedite the development of novel formulations is to repurpose pre-existing drugs that have been approved for the treatment of other medical conditions [20]. Disulfiram (DSF), a drug currently used in the treatment of chronic alcoholism, has been shown to possess anti-tumor activity [21]. DSF can induce apoptosis in some cell lines and reduce cell growth in certain tumours including prostate cancer, breast cancer, lung cancer, leukaemia and cervical adenocarcinoma [21,22,23]. Furthermore, its chemical structure has a high affinity towards copper, which is essential for the tumour angiogenesis processes, and contributes to making DSF selective to cancerous cells, thus sparing healthy cells [24].

(2) Polymer selection—a readily available mucoadhesive polymer that can be utilised in low-cost direct compression of vaginal tablets is desirable. Currently available vaginal dosage forms have several limitations, such as leakage, messiness, and low residence time due to the self-cleansing action of the vaginal tract [25]. Chitosan (CHN) and polyacrylic acid (PAA) are considered mucoadhesive polymers since they have shown an ability to bind to the vaginal mucosa, although remaining attached for different lengths of time [25]. These polymers are polyelectrolytes that dissociate in aqueous solution into either polycations (CHN) or polyanions (PAA) [26,27], which then establish electrostatic interactions in the vaginal environment with the anionic moieties of mucin in the mucus [14]. This interaction does not weaken over time [28] and the polymers would degrade into non-toxic, absorbable subunits that can be metabolised once the drug supply is depleted [8]. CHN produced by the *N*-deacetylation of chitin, is composed of ᴅ-glucosamine and *N*-acetyl-d-glucosamine (deacetylated) randomly linked together by β-(1,4) glycosidic bonds [29]. The percentage of deacetylated glucosamine units (number of ionisable units) along a CHN polymer chain is represented by its degree of deacetylation [30]. CHN exhibits various degrees of deacetylation that contribute to varying molecular weights [31], which are directly proportional to physical properties, such as mucoadhesion [25,28,32]. For mucoadhesive formulations, the molecular weight of CHN should be not so high as to impair hydration and chain entanglement with mucins, but not so low as to give poor adhesion [28]. Thus, a medium molecular weight is a suitable choice [14]. The amino group in CHN has a pKa value of approximately 6.5, which allows it to dissolve in diluted aqueous acidic solvent with a pH lower than 6 [32]. As the formulation to be developed is for vaginal delivery where the pH is 4.2–5 [28], a fast dissolution rate or loss of formulations would be expected from CHN formulations [33,34,35]. PAA exhibits strong hydrogen bonding with the mucin present in the mucosal layer. The hydrophilic nature and cross-linked structure of these polymers make them suitable for controlled drug delivery systems [26]. Since PAA and CHN are differently charged, polyelectrolyte complex formation may reduce the fast dissolution rate observed in CHN-only formulations [35].

(3) Tablet manufacturing—direct compression. Tablets account for nearly 80% of all marketed dosage forms due to economical and stability-related advantages over other dosage forms. Among the tablet-manufacturing processes, direct compression is the simplest and most cost-effective because it only involves blending and compression, as well as many other advantages, including significantly higher manufacturing efficiency and physical and chemical stability [36]. Tablets kept at a smaller size offer a high drug loading capacity, reduce the risk of an inhomogeneous blend or non-uniform active pharmaceutical ingredient (API) (drug) content in finished tablets, and can also improve patient compliance. Most drug loading in direct compression tablet formulations does not exceed 30% because of poor processability, as most drugs have poor flowability and tabletability properties. These properties are critical to ensure the manufacturability of a direct compression tablet formulation [36]. Tablet formulations are typically evaluated to comply with the requirement for tabletability, flowability, friability, disintegration time, tablet ejection force, and dissolution performance [37,38]. If any of these requirements are not met, the formulation is excluded, and a new formulation is designed and evaluated to address identified deficiencies. This process is repeated until an optimized formulation is identified [36].

To date there are numerous formulations that combine 5-FU with DSF, and most bilayer tablets are formulated to provide an immediate drug release from one layer and a sustained or controlled drug release from the other. However, in this study we are exploiting the two layers to physically avoid chemical incompatibilities between the two drugs and to use the two layers to release the drugs concurrently for a synergistic effect in situ. The purpose of this present study is to evaluate the first generation of this intervention and to offer advice for future development.

## 2. Materials and Methods

### 2.1. Materials

Human cervical epidermoid carcinoma (Ca-Ski) cells were acquired from ATCC TCC^®^ (CRL-1550™). Cell culture media and supplements were purchased from GIBCO™ sourced from Thermo Fisher Scientific (Cork, Ireland). Disulfiram (DSF) was obtained from Sigma Aldrich (Wicklow, Ireland). 5-fluorouracil (5-FU) was obtained from Flurorochem (Glossop, UK). Chitosan (medium molecular weight, ≥90% degree of acetylation) was obtained from Glentham (Corsham, UK). Poly(acrylic) acid was obtained from Lubrizol Advanced Materials (Westerlo, Belgium).

### 2.2. Evaluation of Powders (Pre-Compression)

A pre-weighed quantity of powder was placed in a measuring cylinder and the volume occupied was recorded as bulk volume. The bulk density was calculated in g/cm^3^ using the formula in Equation (1). The powder was then placed in a measuring cylinder on a Copley Scientist (Nottingham, UK) tapped density voltmeter and tapped 1000 times. The tapped volume was measured, and the density was calculated in g/cm^3^ using the formula in Equation (2). This procedure was repeated for all the powders used.

(1) $$Bulk\;Density\;\left(BD\right)=\;\frac{Mass\;\left(or\right)weight\;of\;the\;powder\;\left(M\right)}{Volume\;occupied\;\left(V_{0}\right)}$$

(2) $$Tapped\;Density\;\left(TD\right)=\frac{Mass\;\left(or\right)weight\;of\;the\;powder\;\left(M\right)}{Volume\;occupied\;after\;tapping\;\left(V_{t}\right)}$$

The calculated densities were used in computing the compressibility index and Hausner’s ratio using the formulas in Equations (3) and (4) respectively. The flow character of each powder was then evaluated according to Table 1.

(3) $$Compressibility\;Index\;\left(CI\right)=\frac{Tapped\;density\;\left(TD\right)-Bulk\;density\;\left(BD\right)}{Tapped\;Density\;\left(TD\right)}\times 100$$

(4) $$Hausner^{\prime }s\;Ratio\;\left(HR\right)=\frac{Tapped\;Density\;\left(TD\right)}{Bulk\;Density\;\left(BD\right)}$$

### 2.3. Preliminary Formulations and Preparation of Single Blends

Various batches were prepared by varying the weight ratio of polymers to their respective drug, which was kept at 30% *w*/*w*, to identify the most effective formulations to make up the bilayer tablet. Each drug and polymer mixture (from this point on will be referred to as Blend 1 (DSF-PAA) and Blend 2 (5-FU-CHN) were separately prepared by homogeneously mixing the drug, polymer, and sorbitol (bulking agent) in a mortar for 15 min (Table 2 and Table 3). Each blend (150 mg) was then compressed using a 10-mm-diameter die in a single punch tablet press, to make a single blend tablet. Each tablet for both blends was pressed with a pressure of 3 tons for 30 s. An efficient formulation for each blend was determined according to the percentage of in vitro drug dissolution in simulated vaginal fluid (SVF), over 72 h and measured spectroscopically at 217 nm. Other parameters and conditions of the in vitro drug dissolution test were followed according to Section 2.10.

### 2.4. Dissolution of DSF Single Blend Tablet in 2% Sodium Dodecyl Sulphate (SDS) Aqueous Solution

Briefly, 2% (*w*/*v*) of sodium dodecyl sulphate (SDS) was added to distilled water and maintained at pH 4.2 by acetic acid to mimic the vaginal pH. A tablet of Blend 1 was immersed in 900 mL of this dissolution medium and other parameters and conditions of the in vitro drug dissolution test were followed according to Section 2.10. Samples were taken and measured spectroscopically at 217 nm.

### 2.5. Preparation of Bilayer Tablet

Bilayer tablets were prepared by a direct compression procedure involving 2 steps. The formulation of blend 1 (150 mg) was compressed using a 10-mm-diameter die in a single punch machine press, with a pressure of 2 tons for 20 s. The upper punch was raised, and blend 2 formulation (150 mg) was then placed on top of the pressed layer; the 2 layers were then compressed into a bilayer tablet with a pressure of 3 tons for 30 s. In order to ensure good cohesion, the two layers were compressed with different pressures.

### 2.6. Physical Evaluation of Single Blend and Bilayer Tablets

#### 2.6.1. Tablet Uniformity

Twenty tablets were randomly selected and weighed individually using an electric balance, and the thickness was measured using vernier calipers.

#### 2.6.2. Hardness

Ten bilayer tablets were selected randomly and crushed individually between the anvils of the Pharmatron Schleuniger (Thun, Switzerland) Model 6D hardness tester. The energy required to break the individual tablets was recorded and the mean was calculated.

#### 2.6.3. Friability

Twenty tablets were randomly selected, dusted and weighed (*W_0_*) together using an electronic balance. The tablets were then placed into the drum of the friabilator and were rotated at 25 rpm for 4 min. The tablets were dusted and reweighed (*W*). The degree of friability was calculated as percentage of weight loss using the formula in Equation (5), requiring that it be less than or equal to 1% to pass the test.

(5) $$percentage\;loss\;\left(\%\right)=\frac{W_{0}-W}{W_{0}}\times 100$$

### 2.7. Swelling

Swelling study of the various tablets was carried out by submerging tablets in a steel basket into 25 mL of 2% SDS solution (pH 4.2) medium with the temperature maintained at 37 ± 1 °C. Weight of individual tablets was taken prior to the swelling study, *w*_1_. Individual tablets were taken out at time intervals of 2, 4, 6, 8, and 24 h and left to dry in an oven at 60 °C for 24 h before being re-weighed (*w*_2_). Swelling would be indicated by the weight gained by the tablets. Percent hydration (swelling index) was calculated using Equation (6).

(6) $$swelling\;index=\frac{w_{2}-w_{1}}{w_{2}}\times 100$$

### 2.8. Differential Scanning Calorimetry (DSC)

Differential scanning calorimetry (DSC) thermograms were performed using a differential scanning calorimeter (Universal V3.9A TA Instruments, New Castle, DE, USA). Samples (8 mg) were placed into the covered aluminum pans and heated from 25 to 330 °C at a heating rate of 20 °C per minute. The scans were taken under a nitrogen atmosphere. An empty covered aluminum pan was used as the reference.

### 2.9. Scanning Electron Microscopy (SEM)

Scanning electron microscopy (SEM) was performed on a Tescan Mira SEM (Oxford Instruments, Abingdon, UK) using a range of magnifications to evaluate the surface morphology of the tablets and drug using the secondary electrons function. Tablets were snap broken through the transversal plane and cross-sectional areas placed under the microscope. This process was repeated for tablets submerged in liquid nitrogen for 10 min. As a first step, the samples were placed on an aluminum stub and were gold coated using Baltec SCD 005 sputter coater (BAL-TEC Gmbh, Pfäffikon, Switzerland) for 110 s at 0.1 mBar vacuum before observation.

### 2.10. Content Uniformity

Randomly chosen tablets of each formulation batch were weighed accurately, powdered, and 100 mg were dissolved in 100 mL of methanol. Each mixture was shaken at 100 rpm in an incubator overnight and kept at 37 ± 1 °C. Samples (1 mL) were suitably diluted with methanol and analyzed for drug content by a Shimadzu (Kyoto, Japan) UV spectrophotometer at 217 nm and 266 nm.

### 2.11. In Vitro Release Study

Using the paddle method (USP II method) with the rotation speed of 100 rpm and maintained at 37 ± 1 °C, tablets were immersed in 900 mL of 2% SDS solution (pH 4.2). Acetic acid was added into the medium solution to replicate the pH of the cervix at a reproductive age. Samples (5 mL) were withdrawn at 15, 30, 45-, 60-, 90-, and 105-min intervals, then at 2-, 4-, 6-, 8-, 24-, 48-, and 72-h intervals. Samples were assayed using a Shimadzu (Kyoto, Japan) UV spectrophotometer at 217 nm and 266 nm for DSF and 5-FU respectively. Figure S1 shows a scan of UV spectrum of 5-FU, DSF and as a blend from 200 to 400 nm. An equal amount of sample withdrawn was replaced with fresh medium kept at the same temperature to maintain sink conditions. The cumulative percentage of drug released was calculated.

### 2.12. Ex Vivo Mucoadhesion Assessment

The assessment was performed as described by Cazorla-Luna et al. (2019, 2020) with modifications [25,39]. Ewe vaginal mucosa was obtained from Gilligan’s Farm (Four Mile House, Roscommon, Ireland) farming-and-butcher operations and used as a model for ex vivo adhesion. The mucosa was cut into fragments of 75 × 25 mm and fixed on glass slides with cyanoacrylate adhesive. Then, each tablet was placed in the center of the mucosa and pressed with a contact force of 500 g for 10 s. The slides were positioned at an angle of 60° and immersed in 45 mL of simulated vaginal fluid (Table 4), prepared according to Owen and Katz (1999) [40] and, posteriorly incubated at 36.5 °C at 30 rpm until total detachment. The adhesion time was determined by observation of the samples. All assays were performed in triplicate.

### 2.13. Cell Culture Studies

Ca-Ski cells were cultured in humidified incubators (37 °C, 5% CO_2_) in RPMI media supplemented with 10% fetal calf serum (FCS), 1% L-glutamine and 1% penicillin/streptomycin. For experiments, Ca-Ski cells were seeded onto 96 well plates at a density of 7 × 10^3^ cells/well per well and cultured until 60–70% confluence was reached. Media was replaced and cells were treated with varying drug concentrations of DSF and 5-FU independently and in combination. 5-FU and DSF solutions were prepared in dimethyl sulfoxide (DMSO). Stock preparations were made and diluted down at least 100 times in growth media before addition to cells, in order to prevent cytotoxic effects of DMSO. After 48 h, test solutions were removed, and cells were gently washed three times with Dulbecco’s phosphate-buffered saline (DPBS). Cells were incubated with 3-(4,5-Dimethylthiazol-2-yl)-2,5-Diphenyltetrazolium Bromide (MTT) dissolved in DPBS at a final concentration of 0.5 mg/mL. After 3 h the MTT solution was removed. 0.1 mL DMSO was added to each well to dissolve the formazan created by viable cells. Absorbance was read using a Synergy Multi-plate reader at 540 nm. Percentage cell-viability (% Viability) was calculated using absorbance readings of treatment wells against that of the negative control wells.

### 2.14. BrdU Assay

Cells were seeded in 96 well plates at a density of 7 × 10^3^ cells/well (0.1 mL final volume) for 24 h. Cells were then treated with low (40 µM) or high (75 µM) dose DSF; low (10 µM) or high (20 µM) dose 5-FU. To determine additive effect cells were also treated with drug combinations which included: (1) low dose DSF combined with low dose 5-FU; (2) high dose DSF with high dose 5-FU; (3) low dose DSF with high dose 5-FU; and (4) high dose DSF with low dose 5-FU). As a control, a group of cells was kept untreated. Cells were treated for 24 h and subjected to the Cell Proliferation ELISA, BrdU (colorimetric) assay (Roche, Switzerland), following the manufacturer’s protocol. Briefly, cells were incubated for two hours with the BrdU labeling solution, fixed and treated with anti-BrdU-POD solution for 90 min, washed three times with phosphate saline buffer, and incubated with substrate solution for 30 min for the development of color. Absorbance was read at 370 nm in a Synergy™ HTX (BioTek^®^, Winooski, VT, USA) plate reader. Results were plotted as % BrdU positive cells which represents the population of proliferating cells.

### 2.15. Statistical Data Analysis

Continuous variables were expressed as mean ± standard deviation (SD). The change in cell viability and cell proliferation over different doses of DSF or 5-FU was tested by a one-way analysis of variance (ANOVA). If the one-way ANOVA reached statistical significance, pairwise comparisons were performed among different doses of the study drug versus no drug administration (i.e., PBS) by the Fisher’s least significant difference (LSD) test. The effect on cell viability and the BrdU uptake assay combining both DSF and 5-FU at different doses was evaluated by a two-way ANOVA. If the two-way ANOVA reached statistical significance, pairwise comparisons were performed among different dose combination of the study drug versus no drug administration (i.e., PBS) by the Fisher’s LSD test. The effect of dose increase of 5-FU in combination with DSF on the cell viability was depicted using a Karnaugh color map and a heat map. The LD_50_ with 95% confidence interval for DSF and 5-FU was estimated using a non-linear regression by least squares ordinary fit. Goodness of fit was represented by adjusted R square. A *p*-value < 0.05 (two-tailed) was deemed statistically significant. Statistical analyses were performed using STATA-14/MP (StataCorp LP, College Station, TX, USA), GraphPad Prism 7a (GraphPad Software, San Diego, CA, USA), and Microsoft Excel for Mac 2017, Version 15.32 (Microsoft, Redmond, WA, USA).

## 3. Results

### 3.1. Pre-Compression Powder Properties

Table 1 summarises the values of CI and HR in different ranges of values to indicate the different degree of flowability of a specific powder. In this context, the values of CI and HR for the tested powders were evaluated (Table 5). Moreover, 5-FU and CHN showed excellent and fair flowability, respectively, according to their CI and HR values. In contrast, DSF showed poor flowability, and PAA had the lowest degree of flowability.

### 3.2. Determination of Blend 1 and Blend 2 Formulations for the Bilayer Tablet

**Figure 1:**
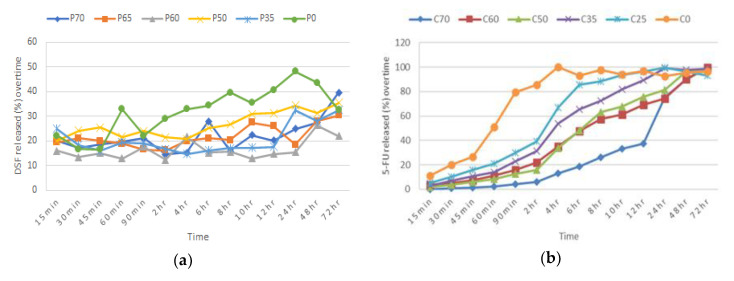
Cumulative percentage of drug released from batches of (**a**) Blend 1 and (**b**) Blend 2.

Batches P0 and C0 from Blend 1 and Blend 2 formulations respectively, are polymer-free and were kept as controls to display the effect of the polymer on the formulations. Both blends, P70 and C70 formulations have shown that the drug-polymer ratio, 30:70, is the most effective for drug release. This ratio was capable of controlling release of both drugs in their respective formulations with the greatest amount released, compared to other batches. Overall, Blend 1 has an erratic drug release profile with a 20.4% (SD 2.99, *n* = 3) initial burst release effect observed within the first 15 min of dissolution (Figure 1a). P70 formulation was able to release 39.6% (SD 3.64, *n* = 3) of DSF within 72 h, which was the highest value as compared to other batches. All batches from Blend 1 formulation showed very low DSF released percentage which highlighted the solubility issue of DSF in the dissolution medium. Blend 2 formulation on the other hand, showed a rather gradual incremental drug release profile across all the batches (Figure 1b). The drug release patterns in this formulation are directly proportional to the amount of polymer. Batches with less than 60% CHN (C50, C35, C25) released more than half of their 5-FU content within the first 6–8 h. C70 formulation was able to sustain more than 60% of its 5-FU content for 12 h and released the remaining amount, 99.4% (SD 1.28, *n* = 3), over the next 60 h.

### 3.3. Dissolution of DSF in 2% SDS Aqueous Solution

**Figure 2:**
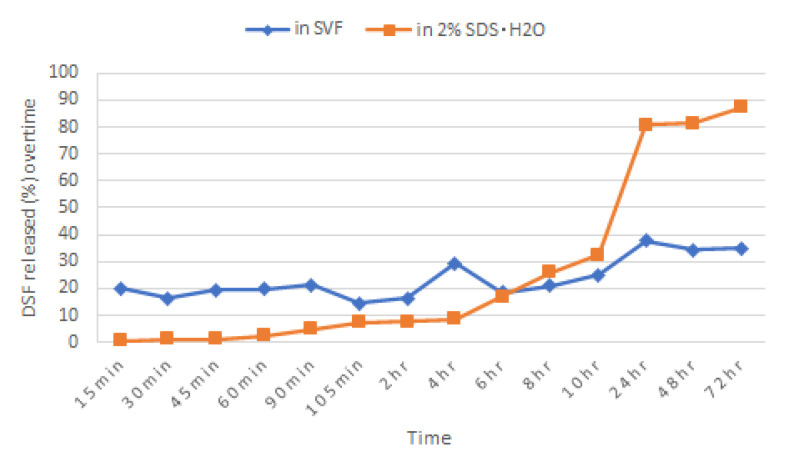
Comparison of cumulative DSF released from P70 formulation in different dissolution media.

Comparison of the dissolution of P70 tablets between SVF and 2% SDS aqueous solution showed that DSF was more soluble in the latter (Figure 2). In this dissolution medium, 87.2% (SD 1.86, *n* = 3) of DSF released was recorded as compared to 39.6% (SD 3.64, *n* = 3) recorded in SVF. Furthermore, the erratic DSF release pattern and the first initial drug release effect was not observed in 2% SDS aqueous solution.

### 3.4. Physical Evaluation of Single Blend Tablets and Bilayer Tablets

The tablets for all formulations were round circular, flat faced and no deformation like chipping, black particles or other stain marks were observed. Blend 1 tablets are off-white/yellowish coloured, Blend 2 tablets were white in colour and these were also observed in the bilayer tablet. Referring to the findings (Table 6). The tablets for all formulations are within the standardised USP limits. The weight, thickness and hardness of all tablet formulations were found to be within the permitted 5% of deviation. The friability for all formulations was well under 1%, with hardness that withstands more than the required force of 4 N and good drug content recorded.

### 3.5. Swelling Test

**Figure 3:**
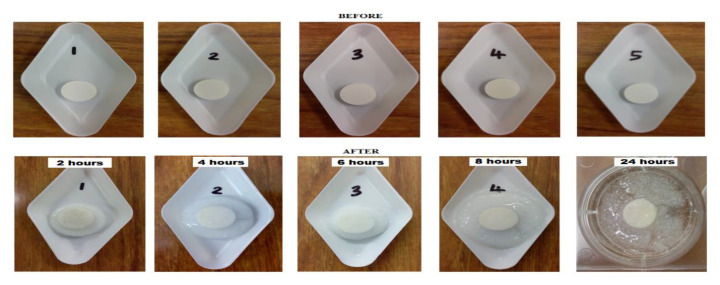
Swelling behavior comparison for bilayer tablets in 2% SDS aqueous solution (pH 4.2), at different time intervals.

**Figure 4:**
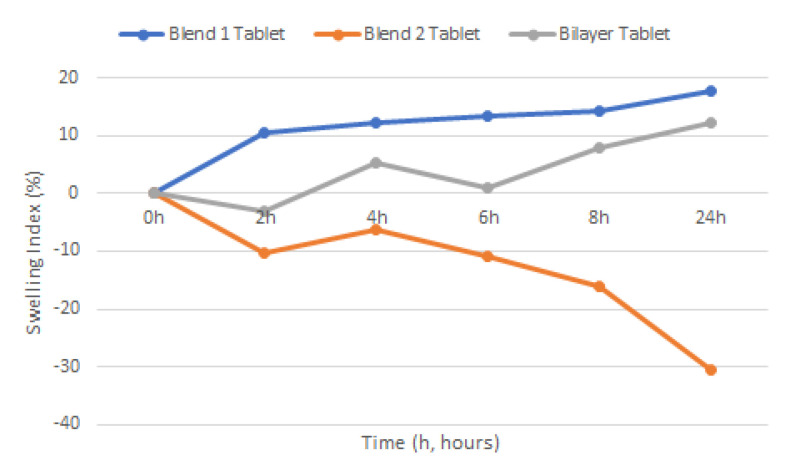
Swelling index of single blend and bilayer tablets.

The swelling test (Figure 3) was evaluated using the calculated swelling index of the different tablets at different time intervals (Figure 4). PAA in Blend 1 demonstrates a more effective swelling characteristic compared to CHN in Blend 2. There was no swelling observed in Blend 2 tablets and the swelling index curve indicated loss of formulation. Presence of the PAA layer in the bilayer tablet formulation was able to reduce the loss of formulation. However, the bilayer tablet did have a mass loss within the first 2 h, though not to the same degree as in Blend 2 tablets. The swelling then began to progress and develop over time.

### 3.6. Tablet Morphology

**Figure 5:**
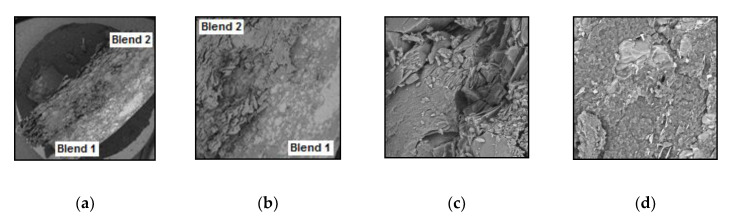
Scanning Electron Micrographs of the bilayer tablet at different magnification. (**a**) Bilayer tablet (×20), (**b**) Bilayer tablet at (×80), (**c**) Blend 1 on bilayer tablet (×1000) and (**d**) Blend 2 on bilayer tablet (×1000).

Figure 5a shows the morphology of the bilayer tablet with a significant difference between the two blends. It can be observed that one blend is more intact (Blend 1) and the other is porous (Blend 2) as shown in Figure 5b. SEM analysis of Blend 2 layer (Figure 5c) showed that both 5-FU and CHN particles preserved their morphology and the drug particles appeared uniformly and finely dispersed as crystalline microaggregates. This was also observed in Blend 1 analysis, however DSF drug particles are heterogeneously mixed with PAA (Figure 5d).

### 3.7. DSC Studies

**Figure 6:**
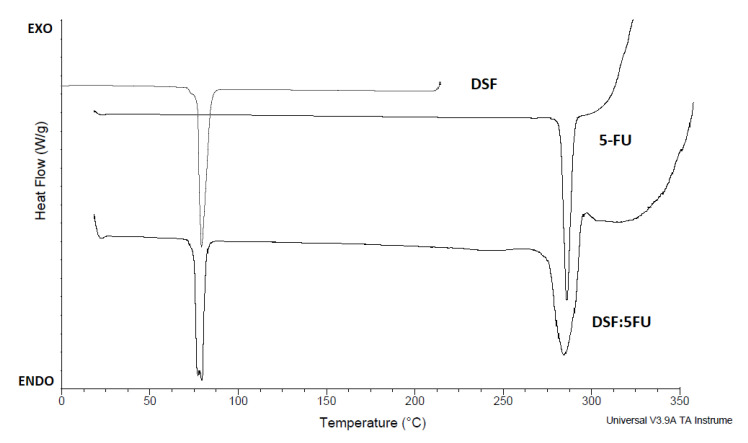
DSC thermogram of single drug substances and in combination.

At a scan rate of 20 ˚C/min, the DSC thermal curves for DSF and 5-FU showed a single sharp endothermic peak at their melting points of 70.8 ± 0.2 °C and 282.4 ± 0.2 °C, respectively (Figure 6). Although there was a slight peak broadening of both drug peaks, there was no significant absence or shifting of the peaks observed when the curve of the 1:1 drug combination was superimposed on single drug curves.

### 3.8. Drug Release Study

**Figure 7:**
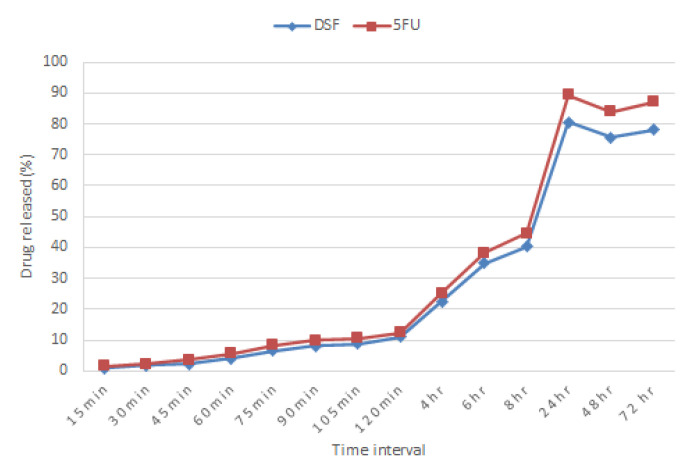
The bilayer release profiles of DSF and 5-FU at 37 ± 1 °C in 2% SDS solution (pH 4.2).

A release of 81% (SD 1.78, *n* = 3) of DSF and 89% (SD 1.78, *n* = 3) of 5-FU over 24 h was observed in the bilayer tablets (Figure 7), and some drug degradation was observed. The percentage of 5-FU released is slightly higher compared to DSF. However, both drugs have a similar release pattern and the release rate is quite compatible.

### 3.9. Cytotoxic Effects of Active Ingredients

**Figure 8:**
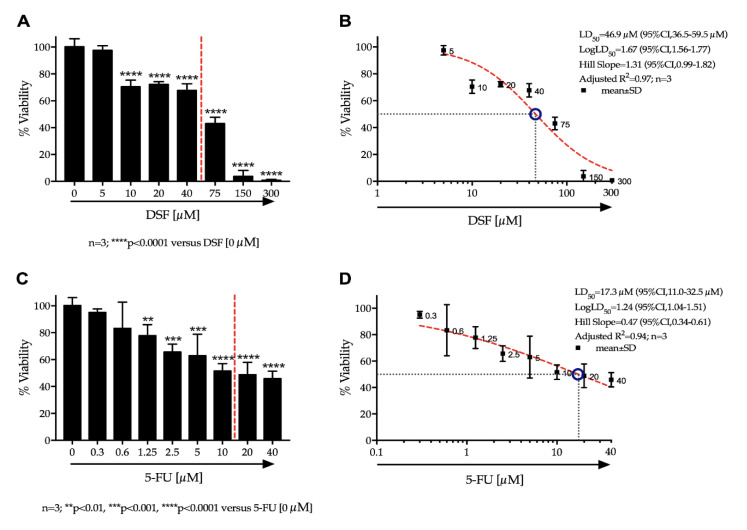
The effects of DSF (Panel **A**, Panel **B**) and 5-FU (Panel **C**, Panel **D**) on the percentage viability of Ca-Ski cells.

Cell viability after drug treatment was determined using the MTT assay. Cell viability decreased in a concentration dependent manner with both drug samples respectively. Significant reduction in viability was observed from 10 μM for DSF (*p* < 0.0001) (Figure 8: Panel A) and 1.25 μM for 5-FU (*p* < 0.05) (Figure 8: Panel C), versus their respective controls. We found that the LD_50_ of DSF (95% confidence interval) in Ca-Ski cells was 46.9 μM (CI: 36.5–59.5 μM) (Figure 8: Panel B). The LD_50_ of 5-FU (95% confidence interval) was determined to be 17.3 μM (CI: 11.0–32.5 μM) (Figure 8: Panel D).

**Figure 9:**
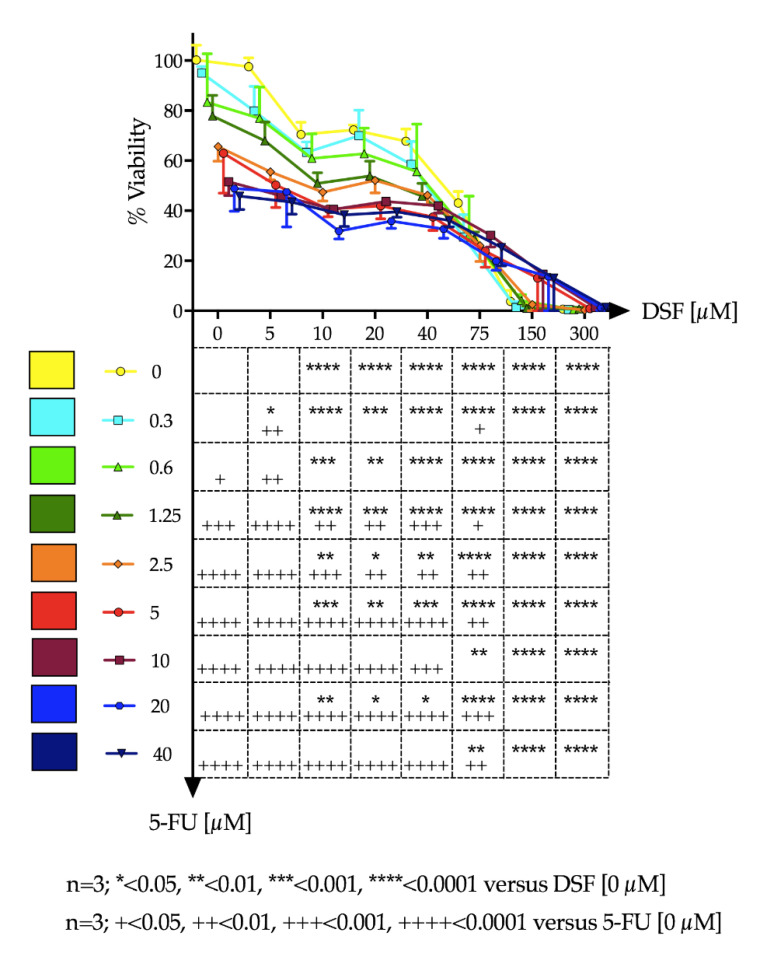
The combined effect of DSF and 5-FU on cell viability of Ca-Ski cells depicted using a Karnaugh color map.

**Figure 10:**
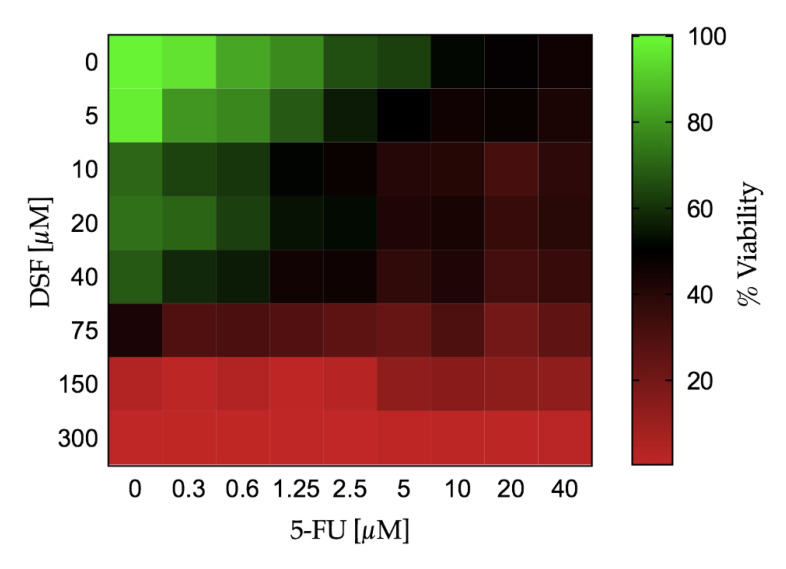
The combined effect of DSF and 5-FU on cell viability of Ca-Ski cells depicted using a heat map.

The effect of dose increase of 5-FU in combination with DSF on cell viability of Ca-Ski cells was determined and depicted in Figure 9 using a Karnaugh color map and in Figure 10 using a heat map. It can be determined from this analysis that 0.3 μM 5-FU and 5 μM DSF have no significant effect on cell viability individually compared to control. However, the combination of these drug dosages has a significant effect on cell viability, suggesting an additive or synergistic effect.

**Figure 11:**
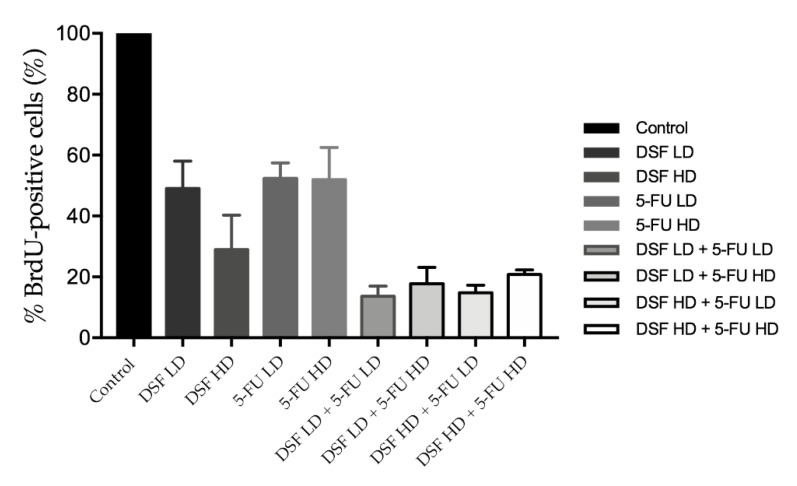
The individual and combined effect of DSF and 5-FU on cell proliferation of Ca-Ski cells determined using a BrdU assay.

To further investigate this combinational effect a BrdU assay was carried out on the drugs individually at a high and low dose (DSF; 40 μM and 75 μM and 5-FU; 10 μM and 20 μM), as well as in combinations of these doses. BrdU-positive cells were normalized to cell number compared to untreated controls to give an accurate representation of the decrease in replication. The results indicate that the drug combinations reduce cellular proliferation significantly more than individually compared to the control at both low and high doses as shown in Figure 11 and Table 7.

## 4. Discussion

### 4.1. Powder Properties

Using the measured densities, the CI and HR were calculated, and these values indicated the degree of flowability and the propensity of a powder to be compressed. These features are essential to ensure well-made tablets and a smooth tableting process [38]. Both are functionally dependent on the interparticle attraction caused by intermolecular forces. When the interparticle attraction is high in a powder, it tends to be cohesive, making it less free-flowing, which is a challenge for compressing. A common way to measure this is by a tapping test which provides the values for HR and CI [37,38]. A low value indicates a little powder cohesiveness, therefore making the powder more free-flowing, and this was observed for both 5-FU and CHN. On the other hand, as the values increases the powder cohesiveness also increases, which results in poor flowability as observed for DSF and PAA; as such these are the most cohesive. The poor flowability and compressibility of a powder can be improved by addition of a glidant, such as talc [41]. Therefore, the drugs and the polymers were paired according to similar flowability characteristics to makeup the two different layers of the formulation. The content of one layer was 5-FU mixed with CHN (Blend 2) and the second layer was DSF mixed with PAA (Blend 1) and talc.

### 4.2. Determination of Blend 1 and Blend 2 Formulations for the Bilayer Tablet

The polymer ratios considered were referred to the studies performed by Sharma et al. (2006) and Fitaihi et al. (2018), for PAA and CHN respectively [42,43]. The cumulative percentage drug release of both polymers showed that the drug-polymer ratio of 30:70 (*w*/*w*) is an effective ratio and exhibits a controlled drug release profile over the span of 72 h. With regard to the short life span of 5-FU (< 30 min), C70 formulation was appreciatively able to sustain the stability of 5-FU in the tablet and gradually released up to 100% of its dose content. Furthermore, there were no initial burst release effects observed for the C70 formulation. On the other hand, even though erratic, all batches of Blend 1 formulations showed significant increments for DSF release over time. The P70 formulation has shown to release the most DSF within the 72 h. C0 and P0 formulations were controlled with polymer-free formulations. The high release percentage of the C0 formulation has proven that the controlled release of 5-FU was in response to the CHN added in the other batches of Blend 2 formulations. This is also the case observed for Blend 1 formulations. However, the P0 formulation has also highlighted the low solubility of DSF in SVF.

### 4.3. Dissolution of P70 Tablets in 2% SDS Aqueous Solution

Since P70 is the formulation that released the greatest amount of DSF, this batch was chosen to compare the dissolution of DSF between SVF and 2% SDS aqueous solutions. Many studies, such as those of Boyd et al. (2014) and Tiez et al. (2019), have acquired in vitro drug release data for their disulfiram-loaded devices in a 2% SDS solution [7,44]. At this point the in vitro method was mainly designed for evaluation purposes, namely, to ensure a specific release pattern within a certain time frame and to discriminate between good and bad batches, rather than for simulating in vivo conditions. DSF solubility was improved significantly using 2% SDS aqueous solution as the dissolution medium. Furthermore, there is an absence of initial burst release effect and the percentage of drug release increments are much more gradual and compatible with the 5-FU release pattern.

### 4.4. Physical Evaluation of Tablets

According to the US Pharmacopeia (USP), the weight and thickness should not deviate more than ± 5% of its mean. The tablets from all formulations fell within this parameter. However, blend 1 showed the largest variation which could be caused by inconsistencies during the weighing process due to the powder’s poor flowability. The SD values relating to the thickness for all formulations were relatively low which reflects on the consistency of the tablet pressing method. The resistance of tablets to withstand abrasion or chipping during packaging, handling, and shipping depends on the hardness and friability. The mean energy reported for all formulations can withstand more than the minimum crushing force indicated for a tablet which is 4 N, according to the USP. Furthermore, all formulations did not lose more than 1% of their weight (*n* = 20) in the friability test, which is satisfactory to the limit specification of USP.

### 4.5. Swelling Test

Swelling can occur when tablets are subjected to hydration, and it is an important assessment as it is directly connected to mucoadhesion ability. Shortly after swelling in the presence of water a gel layer will be produced, which will cause mucoadhesion by forming a weak bond to a given mucosal membrane. The swelling should not lead to the detachment of small fragments of tablets and the tablet should however remain intact over a period of time. From the observation, the ability of PAA to swell extensively is facilitated by the carboxylic groups in the polymer chain, which are readily ionizable and strongly associate with water molecules. CHN on the other hand, readily dissociates in dilute aqueous acidic solution which makes it soluble in this dissolution medium (pH 4.2). Therefore, loss of formulation was reported for Blend 2 tablets. In the bilayer tablet, where both PAA (anionic) and CHN (cationic) are present in one formulation, this creates a complex that temporarily forms a cross-linking network between these polymers and stabilizes their swelling mechanism [35]. PAA is also called a super-absorbent, and has the ability to absorb many times its weight in water [45]. However, if the hydration level is too high, the mucoadhesion property is expected to be reduced due to the competition between water molecules and the active groups in the mucin chains of the biological membrane to bind to the polymer functional groups [43,46]. Therefore, the presence of CHN in the formulation, reduces swelling of PAA and the risk of overhydration. In addition, the polymer-polymer based polyelectrolyte complex formation was able to reduce the solubility of CHN in the acidic medium and make it more stable in the vaginal environment. Another important observation of this study was that the two layers did not detach from one another, which shows a good potential for retention of the whole formulation, thus prolonging bioavailability of the drugs in the target site.

### 4.6. SEM Study

To assess the surface morphology of the bilayer formulation, SEM studies were carried out. SEM photomicrographs showed homogeneous (Blend 2 layer) and heterogeneous (Blend 1 layer) mixtures of drugs and excipients. SEM analysis of Blend 1 showed a compact and an uneven structure in this mixture, which reflects the properties of the powders. Earlier, both DSF and PAA were found to have low flowability, due to their high intraparticle attraction, which means that they tend to clump together. The drug particulate clumping appears as obvious white colored clusters with various sizes throughout the Blend 1 layer. This would also be the reason as to why the DSF release profile was erratic, due to the dissolution of different sizes of DSF clumps released from the polymer. The analysis of Blend 2 showed a more porous morphology, which was expected due to the presence of CHN [47], and was homogeneously dispersed with 5-FU crystalline microaggregates. These observations indicated that the drugs and polymers preserved their individual morphology even after the tablet pressing process.

### 4.7. DSC Study

Potential physical and chemical interactions between drugs and excipients can affect the chemical nature, the stability and bioavailability of drugs and, consequently, their therapeutic efficacy and safety [48]. In order to exclude possible interactions between the drugs and polymers used, DSC analysis was conducted. DSC is a rapid analytical technique commonly used for evaluating interactions in formulations through the appearance, shift, or disappearance of endo- or exothermic effects and/or variations in the relevant enthalpy values [48]. In the DSF: 5-FU (1:1 *w*/*w*) thermal curve the characteristic endotherms of both drugs were present, and there were no extra-thermal effects observed. However, there was slight peak broadening and a reduction in both peak sizes, which indicates a typical drug–drug solid–solid interaction. Nonetheless, when superimposed on the thermal curves of pure DSF and 5-FU, it is conclusive that there were no interactions or physical incompatibility between these two drugs.

### 4.8. In Vitro Drug Release Study of Bilayer Tablets

The vaginal pH of women in the reproductive age is acidic with pH 4-5 [43], therefore, the study was carried out with a medium pH of 4.2 and maintained at 37 ± 1 °C. It was observed that there was no initial burst release effect for either drug from the bilayer tablet. Both layers exhibited almost similar drug release profiles with only approximately 40% of the drug contents released within the first 8 h, and a surge of drug release by 24 h. The release profile for both drugs was quite compatible with each other as the drugs were released concurrently with no one drug being released at a rate greater than 2–9% over the other at any specific time. Tablet dissolution is strongly affected by swelling and solvent penetration into its matrix [49]. This was observed in the drug release which corresponds to the swelling study. The swelling study started at the second hour which was represented by a negative value. This resembles the drug release of no more than 10% within the first 2 h. It is after the 2 h mark that drug was released at a higher rate which corresponds to the increasing swelling index over the same period.

### 4.9. Ex Vivo Mucoadhesion Assessment

The mucoadhesion test was performed to establish the mucoadhesion properties of the tablets to vaginal mucosa. Simulated vaginal fluid (SVF) was used to mimic the conditions of the cervix to which the tablets would be exposed in a clinical setting [50]. The tablet remained attached to mucosa for 28 h without disintegrating. This characteristic is essential to avoid losses by fragmentation [25] and to ensure a correct drug release.

### 4.10. Cytotoxic Effects of Active Ingredients

The two drugs were tested for potential cytotoxic effects on a cervical cancer cell line. Effects were measured alone or in combination. 5-FU is a common treatment for malignancies which disrupts DNA synthesis by two modes of action, first by inhibiting cell proliferation via direct incorporation into RNA causing abnormal base pairing. Secondly, it binds thymidylate synthase, blocking the conversion of deoxyuridine monophosphate to deoxythymidine monophosphate, which is essential to DNA synthesis [2,15]. DSF has been used for several decades as a deterrent in the treatment of alcohol addiction as it inhibits the enzyme aldehyde dehydrogenase (ALDH) which is responsible for metabolizing alcohol and has been shown to possess anti-tumor activity by inducing apoptosis. Results show that as drug concentration increased cell viability decreased. Combination studies suggest that the drugs may show a synergistic effect. At a dose of 0.3 μM, 5-FU had no significant effect; DSF at a concentration of 5 μM also had no significant effect on cell viability. However, when both doses were administered in combination, a significant reduction in viability was observed. This is extremely beneficial as it suggests that much lower concentrations of the two drugs can be used to achieve a reduction in cell viability, compared to the concentrations required for the drugs when used individually. This could reduce potential side effects often observed by these two anticancer drugs. These results also support the advantages of the use of bi-layer tablets.

To gain further insight into this interaction a BrdU assay was carried out on the drugs individually at a higher and lower dose with respect to the LD50 (DSF: 40 μM and 75 μM; and 5-FU: 10 μM and 20 μM). The results of the BrdU incorporation assay showed significant reduction in proliferating cells when all combinations were administered compared to control as well compared to the drugs administered individually. This result may indicate that the combination of treatments interferes more efficiently with DNA synthesis in the cancerous cells than the drugs would in isolation. This is extremely beneficial as lower doses of both drugs can be administered in combination to achieve a more significant effect. This is important as a main goal of combinatorial drug administration is to reduce the required dosages. The results also show there is no benefit of administering higher drug dose combinations as the lower dose combinations achieve the same effect on cell viability and growth.

## 5. Conclusions

This study was carried out to develop a vaginal bilayer tablet to be used in conjunction with the cervical cancer treatments available in low resource countries. A vaginal tablet is a very common dosage form that is easy to manufacture, easy to place in the vagina, and has a low manufacturing cost, making them ideal for use in low resource countries. The vaginal tablet developed in this study complies with all the physical evaluation parameters required, with satisfactory mucoadhesion suitable for vaginal drug delivery. The drug combination of DSF and 5-FU evaluated using Ca-Ski cells has shown benefits in combining low-cost generic compounds with additive and potentially synergistic effects at low concentrations. The bilayer tablet can be a promising strategy as it can avoid chemical incompatibilities, deliver drugs with different mechanisms of action for increased efficacy, and release both drugs concurrently at a compatible rate for synergistic effect at target site.

## Figures and Tables

**Table 1:** Indication of flow character by the compressibility index and Hausner’s ratio values.

Compressibility Index (CI)	Flow Character	Hausner’s Ratio (HR)
1–10	Excellent/Very free flow	1.00–1.11
11–15	Good/Free flow	1.12–1.18
16–20	Fair	1.19–1.25
21–25	Passable	1.26–1.34
26–31	Poor/Cohesive	1.35–1.45
32–37	Very poor/Very cohesive	1.46–1.59
>38	Very very poor/non-flow	>1.60

**Table 2:** Composition of Blend 1 * tablets **.

Ingredients (mg/Tablet)	P70 **	P65 **	P60 **	P50 **	P35 **	P0 **
DSF	45	45	45	45	45	45
PAA	105	97.5	90	75	52.5	0
Sorbitol	0	7.5	15	30	52.5	105
Total	150	150	150	150	150	150

* Blend 1 indicates the mixture of disulfiram (DSF) and polyacrylic acid (PAA). ** Each batch contains 1% Talc.

**Table 3:** Composition of Blend 2 * tablets.

Ingredients (mg/Tablet)	C70	C60	C50	C35	C25	C0
5-FU	45	45	45	45	45	45
CHN	105	90	75	52.5	37.5	0
Sorbitol	0	15	30	52.5	67.5	105
Total	150	150	150	150	150	150

* Blend 2 indicates the mixture of 5-fluorouracil (5-FU) and chitosan (CHN).

**Table 4:** Simulated vaginal fluid (SVF) composition.

Composition in Water	mg·mL^−1^
Sodium chloride	3.51
Potassium hydroxide	1.40
Calcium hydroxide	0.22
Bovine serum albumin	0.02
Lactic acid	2.00
Acetic acid	1.00
Glycerol	0.16
Urea	0.40
Glucose	5.00
pH	4.20

**Table 5:** Degree of flowability of each powder determined by their calculated CI and HR values.

Powder	Compressibility Index	Hausner’s Ratio	Degree of Flow Ability
5-FU	4.00	1.042	Excellent/Very free flow
DSF	26.00	1.351	Poor/Cohesive
CHN	20.00	1.250	Fair
PAA	34.00	1.515	Very poor/Very cohesive

**Table 6:** Physicochemical properties of single blends and bilayer tablets.

Formulation	* Weight (mg) ^a^	Weight Deviation (%) ^a^	* Thickness (mm) ^a^	Thickness Deviation (%) ^a^	Hardness (N) ^b^	Friability (%) ^a^	* Drug Content (%) ^c^
**Blend 1**	284.99 ± 6.38	−1.9 to 1.8	3.60 ± 0.05	−2.5 to 2.5	4.38 ± 0.41	0.52 ± 0.07	97.3 ± 0.70
**Blend 2**	298.05 ± 4.70	−4.6 to 3.1	3.64 ± 0.05	−2.5 to 1.7	4.4 ± 0.25	0.10 ± 0.10	98.3 ± 0.51
**Bilayer**	297.32 ± 4.52	−2.4 to1.8	3.67 ± 0.05	−3.8 to 1.7	4.37 ± 0.40	0.23 ± 0.04	97.3 ± 1.00 (DSF)
							98.5 ± 0.92 (5-FU)

* Each value represents mean ± SD (standard deviation); ^a^ *n* = 20; ^b^ *n* = 10, ^c^ *n* = 3.

**Table 7:** Multiple comparisons between low and high dose DSF versus low and high dose 5-FU and different combination doses of DSF and 5-FU. The results highlighted in bold *italics* report a lower cell replication by administering the 2 combined drugs at the lowest doses compared with both the single low and high dose of DSF or 5-FU. DSF LD = 40 μM; DSF HD = 75 μM; 5-FU LD = 10 μM; 5-FU HD = 20 μM.

Variable 1	Variable 1	*p*-Value
Control	DSF LD	< 0.0001
Control	DSF HD	< 0.0001
Control	5-FU LD	< 0.0001
Control	5-FU HD	< 0.0001
Control	DSF LD + 5-FU LD	< 0.0001
Control	DSF LD + 5-FU HD	< 0.0001
Control	DSF HD + 5-FU LD	< 0.0001
Control	DSF HD + 5-FU HD	< 0.0001
DSF LD	DSF HD	< 0.0001
DSF LD	5-FU LD	0.335
DSF LD	5-FU HD	0.396
DSF LD	DSF LD + 5-FU LD	***< 0.0001***
DSF LD	DSF LD + 5-FU HD	< 0.0001
DSF LD	DSF HD + 5-FU LD	< 0.0001
DSF LD	DSF HD + 5-FU HD	< 0.0001
DSF HD	5-FU LD	< 0.0001
DSF HD	5-FU HD	< 0.0001
DSF HD	DSF LD + 5-FU LD	***< 0.0001***
DSF HD	DSF LD + 5-FU HD	0.0020
DSF HD	DSF HD + 5-FU LD	0.0001
DSF HD	DSF HD + 5-FU HD	0.0214
5-FU LD	5-FU HD	0.9074
5-FU LD	DSF LD + 5-FU LD	***< 0.0001***
5-FU LD	DSF LD + 5-FU HD	< 0.0001
5-FU LD	DSF HD + 5-FU LD	< 0.0001
5-FU LD	DSF HD + 5-FU HD	< 0.0001
5-FU HD	DSF LD + 5-FU LD	***< 0.0001***
5-FU HD	DSF LD + 5-FU HD	< 0.0001
5-FU HD	DSF HD + 5-FU LD	< 0.0001
5-FU HD	DSF HD + 5-FU HD	< 0.0001
DSF LD + 5-FU LD	DSF LD + 5-FU HD	0.2280
DSF LD + 5-FU LD	DSF HD + 5-FU LD	0.7383
DSF LD + 5-FU LD	DSF HD + 5-FU HD	0.4130
DSF LD + 5-FU HD	DSF HD + 5-FU LD	0.3811
DSF LD + 5-FU HD	DSF HD + 5-FU HD	0.3886
DSF HD + 5-FU LD	DSF HD + 5-FU HD	0.0852

## Supplementary Materials

The following are available online at https://www.mdpi.com/1999-4923/12/12/1185/s1, Figure S1: Scan of UV spectrum of 5-FU, DSF and as a blend from 200 to 400 nm.
