# Supplementary material for: A Bilayer Vaginal Tablet for the Localized Delivery of Disulfiram and 5-Fluorouracil to the Cervix
Source: Pharmaceutics. 2020 Dec 6;12(12):1185. doi: 10.3390/pharmaceutics12121185 (PMC7762309; doi:10.3390/pharmaceutics12121185)
Supplement: Supplementary file 1 [file pharmaceutics-12-01185-s001.pdf]

pharmaceutics

MDPI

A Bilayer Vaginal Tablet for the Localized Delivery of Disulfiram and 5-Fluorouracil to the Cervix

Ismin Zainol Abidin, Emanuele Rezoagli, Bianca Simonassi-Paiva, Gustavo Waltzer Fehrenbach, Kevin Masterson, Robert Pogue, Zhi Cao, Neil Rowan, Emma J. Murphy and Ian Major

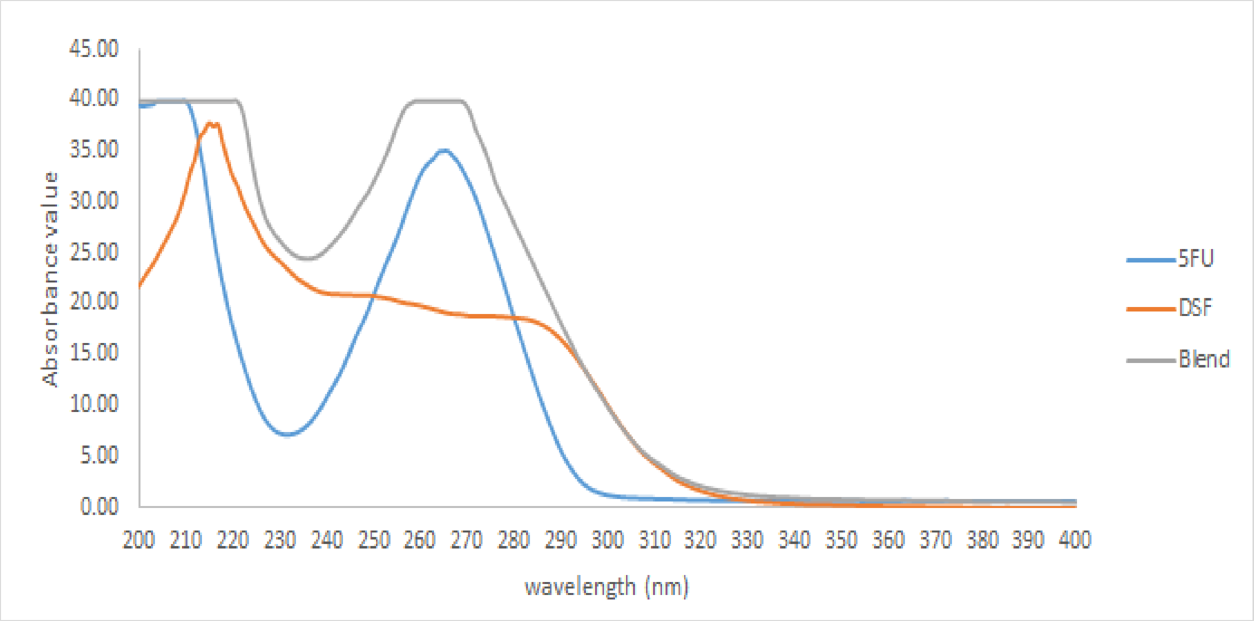

Figure S1. Scan of UV spectrum of 5-FU, DSF and as a blend from 200 to 400 nm.
